# Assessment of mortality risks due to a strong cold spell in 2022 in China

**DOI:** 10.3389/fpubh.2023.1322019

**Published:** 2023-12-07

**Authors:** Wanci Wang, Yuxia Ma, Pengpeng Qin, Zongrui Liu, Yuhan Zhao, Haoran Jiao

**Affiliations:** ^1^College of Atmospheric Sciences, Key Laboratory of Semi-Arid Climate Change, Ministry of Education, Lanzhou University, Lanzhou, China; ^2^Liaoning Provincial Meteorological Bureau, Shenyang, China

**Keywords:** cold spell, temperature change, mortality, excess death, health risks

## Abstract

**Background:**

With the intensification of global climate warming, extreme low temperature events such as cold spells have become an increasingly significant threat to public health. Few studies have examined the relationship between cold spells and mortality in multiple Chinese provinces.

**Methods:**

We employed health impact functions for temperature and mortality to quantify the health risks of the first winter cold spell in China on November 26^th^, 2022, and analyzed the reasons for the stronger development of the cold spell in terms of the circulation field.

**Results:**

This cold spell was a result of the continuous reinforcement of the blocking high-pressure system in the Ural Mountains, leading to the deepening of the cold vortex in front of it. Temperature changes associated with the movement of cold fronts produced additional mortality risks and mortality burdens. In general, the average excess risk (ER) of death during the cold spell in China was 2.75%, with a total cumulative excess of 369,056 deaths. The health risks associated with temperatures were unevenly distributed spatially in China, with the ER values ranging from a minimum of 0.14% to a maximum of 5.72%, and temperature drops disproportionately affect southern regions of China more than northern regions. The cumulative excess deaths exibited the highest in eastern and central China, with 87,655 and 80,230 respectively, and the lowest in northwest China with 27,474 deaths. Among the provinces, excess deaths pronounced the highest in Shandong with 29,492 and the lowest in Tibet with only 196.

**Conclusion:**

The study can provide some insight into the mortality burden of cold spells in China, while emphasising the importance of understanding the complex relationship between extreme low temperature events and human health. The outcomes could provide valuable revelations for informing pertinent public health policies.

## 1 Introduction

In the context of global warming, the frequency and intensity of extreme weather events such as cold spells and heat waves are increasing (1–3). The association between climate and public health has become a pressing issue that will likely result in more health crises in the future (4). According to the Sixth Assessment Report of IPCC (5), climate change may exacerbate the occurrence of extreme weather events, which leads to the increase in the incidence and mortality of climate sensitivity diseases in many places in the world, especially in extremely low temperature weather condition (6, 7). The occurrence of climatic events will cause relevant sensitive diseases to be more affected. Due to the aging of the population, the proportion of vulnerable groups in society is also increasing, which further the health risks of extreme weather are exacerbated (8). It’s imperative to investigate the connection between climatic and environmental factors and human health.

Temperature is a crucial factor that influences human health (9, 10). Extensive studies have consistently demonstrated a non-linear relationship between temperature and health outcomes, with exposure-response curves for morbidity or mortality in populations typically having a “U,” “V” or “J” shape (9, 11–13). A study encompassing 15 European cities revealed that a 1°C drop in temperature resulted in an increase of 1.35, 1.72, 3.30, and 1.25% in daily natural deaths, cardiovascular diseases, respiratory diseases, and cerebrovascular diseases, respectively (14). Intriguingly, low temperature contributes to a greater proportion of cardiovascular disease-related deaths compared to heat-related conditions. An extensive study from 15 major cities in China from 2007 to 2013 elucidated that 15.8% of cardiovascular mortality was attributable to low temperatures, whereas a mere 1.3% could be attributed to high temperatures (15).

The cold spell is a typical extreme low temperature event. In recent years, extreme cold weather events have been creeping up in many regions of the world and are becoming increasingly fierce. For instance, during the strong cold spell in February 2021, North America experienced record-breaking low temperatures that resulted in 100 deaths and left 5.5 million households suffering from power outages (16). Similarly, in December 2022, an “epic cold spell” swept through the United States, causing over 60 deaths (17). In different studies, the definition of cold spells is inconsistent, leading to differences in analysis results. Wang et al. (18) defined the period when the temperature was lower than the 5th percentile and lasted more than 2 days as a cold spell. The study found that the overall cumulative excess risk (CER) of non-accidental deaths during cold weather in China from 2006 to 2011 was 28.2% (95% CI: 21.4, 35.3%), and the impact was more severe in southern China compared to the northern region. Sun et al. (19) use the definition of a cold spell with temperature below the 5th percentile and lasting more than 7 days. Compared to non-cold spell days, the risk of non-accidental mortality, circulatory mortality, and respiratory mortality increased by 17.4% (95% CI: 15.8, 19.0%), 20.8% (95% CI: 18.8, 23.0%), and 22.7% (95% CI: 19.5, 25.9%) respectively on cold spell days.

To gain a deeper insight into the health risks associated with cold spells, in the current study, we focused on the initial cold spell event of winter in China that occurred on November 26^th^, 2022. This particular cold spell was characterized by rapid spread, wide-reaching impacts, complex precipitation and snow phases, and significant temperature drops across over 30 provinces. This can be considered as a typical outbreak of cold spell weather process. A correlation between the temperature reduction of the cold spell and mortality was established, and then it could provide a quantitative estimation of the health risks associated with this specific cold wave event. The findings will enhance our comprehension of the connection between extreme cold events and mortality rates in various regions, as well as aid in the development of effective adaptation strategies to mitigate the adverse effects of future extreme cold events.

## 2 Data and methods

### 2.1 China meteorological data set

Daily meteorological station data were collected from China Meteorological Data Service Centre,^1^ including average temperature, atmospheric pressure, sea level pressure, relative humidity, precipitation, wind direction, and wind speed before and after the onset of the cold spell. These data are sourced from national-level surface meteorological stations managed and subject to quality control by the China Meteorological Administration. The data are primarily collected from the major cities in the country, making it representative and reliable.

The reanalysis data were obtained from ERA5, the fifth generation of ECMWF atmospheric reanalysis global climate data with high temporal and spatial resolution. It provides hourly estimates of atmospheric, terrestrial, and oceanic climate variables, including 137 layers of atmospheric data. The selected time period ranges from 26 November to 5 December 2022 and consists of 2 m temperature, 500 hPa geopotential height, temperature, and sea level pressure variables. The data have a horizontal resolution of 0.25° × 0.25° and a temporal resolution of 1 h.

### 2.2 Population and baseline mortality data

Highly accurate population data in 2019, precise to 1 × 1 km^2^, were provided by the Resource and Environment Science and Data Center.^2^ These data are based on multiple population-relevant characteristics, including land-use type, night light brightness, and settlement density, ensuring accurate representation of the spatial distribution of China’s population. The population distribution of China in 2022 is calculated using the annual population growth rate of China’s population since 2019 (Supplementary Table S1).

The baseline mortality data for each province were gathered from the National Bureau of Statistics,^3^ including various indicators such as the national economy, population, education, and health. The data is reliable, complete and accurate, and it can reflect diverse social and economic activities across the country and. However, the mortality rate data used in the study is only counted to 2021, as the latest data for 2022 has not yet been updated by the country.

### 2.3 Definition of a cold spell

A cold spell was defined as below by the Central Meteorological Observatory: when the temperature drops were more than 8°C within 24 h or 10°C in 48 h or 12°C in 72 h and the minimum temperature is below 4°C.^4^

### 2.4 Calculation of excess deaths

Firstly, the relative risk (*RR*) of the temperature reduction of cold spell is calculated using the following equation:

$RR_{i}=\exp\left(\beta \;.\;\Delta x_{i}\right)$ (1).Where β is the exposure response relationship coefficient, which represents the additional mortality risk per 1°C decrease in temperature. The β value reference is chosen as 0.21% (20), and $\Delta x_{i}$ is the difference between the maximum and minimum of the daily mean temperature at grid i during the cold spell.


(2) $$ER_{i}=RR_{i}-1$$

$ER_{i}\;$ is the excess risk ( ER ) of mortality related to temperature at grid i , it reflects the variation in the ER of mortality associated with every 1°C change in temperature.

We evaluated the mortality burden attributed to this cold spell in China using the following formula (21):

(3) $$\Delta Mortality=Y_{i}\;.\;ER_{i}\;.\;POP_{i}$$

Where ΔMortality indicates the excess mortality rate related to temperature, $Y_{i}\;$ is the baseline mortality rate, and $POP_{i}$ is the total population exposed to low temperature.

As the spatial resolution of the population data differs from that of the 2 m temperature, we used geographic information system ( GIS ) technology to resample the population data raster to 0.25° using bilinear interpolation. The mortality attributed to low temperature exposure in each grid in China was subsequently estimated by utilizing the combined exposure-response coefficient. After calculating the mortality in each grid separately, subdivisional statistics in each province by using the spatial analyst tool in ArcMap (version 10.8). We have also divided China into seven geographical regions based on variations in climate and population, encompassing Northeast China, including Heilongjiang, Liaoning, and Jilin; North China, consisting of Beijing, Tianjin, Hebei, Shanxi, and Inner Mongolia; Northwest China, comprising Shaanxi, Gansu, Ningxia, Xinjiang, and Qinghai; East China, encompassing Jiangsu, Zhejiang, Anhui, Shandong, and Shanghai; Central China, consisting of Henan, Hubei, Hunan, and Jiangxi; Southwest China, encompassing Sichuan, Tibet, Guizhou, Yunnan, and Chongqing; and South China, comprising Fujian, Guangdong, Guangxi, Hainan, and Taiwan. Additionally, to verify the rationality of the ERA5 reanalysis data, we employed two commonly used indexes to evaluate and compare it with observation station data. The results can be found in Supplementary Figure S1 in the Appendix.

## 3 Results

### 3.1 Cold spell process

**Figure 1:**
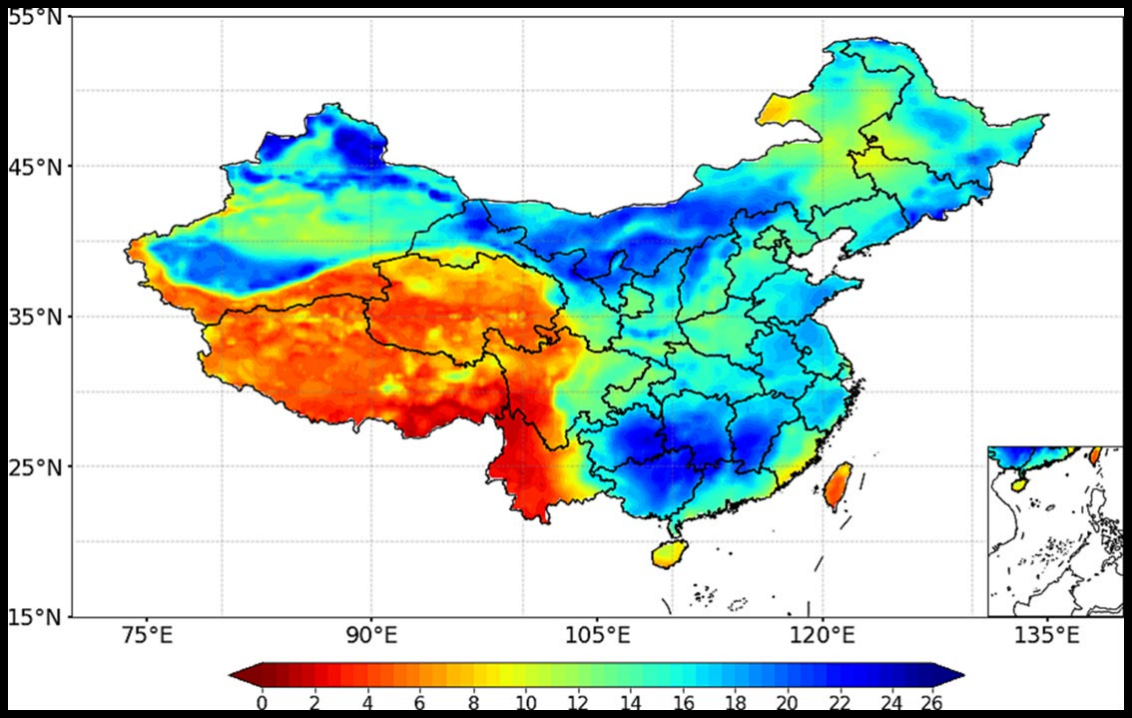
The temperature drops from November 26^th^ to December 5^th^, 2022.

As illustrated in the Figure 1, the cold spell from November 26^th^ to December 5^th^, 2022 had caused a significant cooling effect across most parts of China, with the maximum temperature drop between 8°C and 16°C, which was particularly pronounced in Xinjiang, Gansu, Inner Mongolia, Shanxi, Guizhou, Hunan, Jiangxi, and other provinces where temperature drops exceeded 20°C. The temperature showed the decreasing trend evidently in the northwestern, eastern as well as in southern China, where the big cities with dense population are also greatly affected and expected to pose greater health risks. The temperature drop exceeded 16°C over a national area of 2.15 million square kilometres (about 22% of the country). According to the statistics from the China Meteorological Administration, certain regions in northwest, northern, eastern China, western and southern Jiangnan, as well as north-central China experienced the earliest temperature drops in recorded history, reaching a level of extremity. In addition, we summarized the descriptive statistics for the major cities in the 14 provinces with the highest range of temperature dropping process (see Table 1). It is evident that despite the notable differences in average temperatures between the north and south, all of these regions have experienced temperature drop exceeding 15°C, as well as significant increases in sea level pressure before and after the cold spell. The Chinese meteorological authorities have assessed the overall intensity of the cold spell that swept through most of China to be the fifth strongest one on record for the same period in November.

**Table 1:** Statistics of meteorological elements in major cities affected by the cold spell.

City	Temperature (°C)		Mean Maximum wind speed (m/s)	Mean relative humidity (%)	Sea level pressure (hPa)	
	The reduced temperature of the process	Mean			Min	Max
Hohhot	17.33	−8.99	4.74	35.13	1009.20	1052.90
Lüliang	19.71	−4.83	5.25	42.26	1005.90	1052.10
Nanjing	17.57	8.28	6.28	65.59	1002.40	1036.20
Nanchang	18.36	6.51	8.07	84.44	1007.70	1035.90
Hefei	17.35	7.56	4.09	70.77	1006.60	1040.40
Hangzhou	16.64	9.66	6.02	87.81	1007.80	1037.00
Nanning	18.93	17.65	7.62	88.06	1006.70	1027.80
Qingdao	19.47	5.11	11.09	61.33	1010.00	1041.10
Changsha	19.05	8.55	8.74	83.31	1005.60	1037.60
Guiyang	21.77	10.12	7.50	80.11	998.10	1034.10
Urumqi	16.27	−13.80	3.78	68.44	1012.80	1057.00
Lanzhou	15.87	−4.97	3.32	54.69	1002.80	1045.90
Yinchuan	16.78	−4.72	3.97	38.78	1003.80	1052.10
Yulin	19.61	−6.14	5.78	38.44	1007.80	1053.60

### 3.2 Analysis of the causes of the cold spell

**Figure 2:**
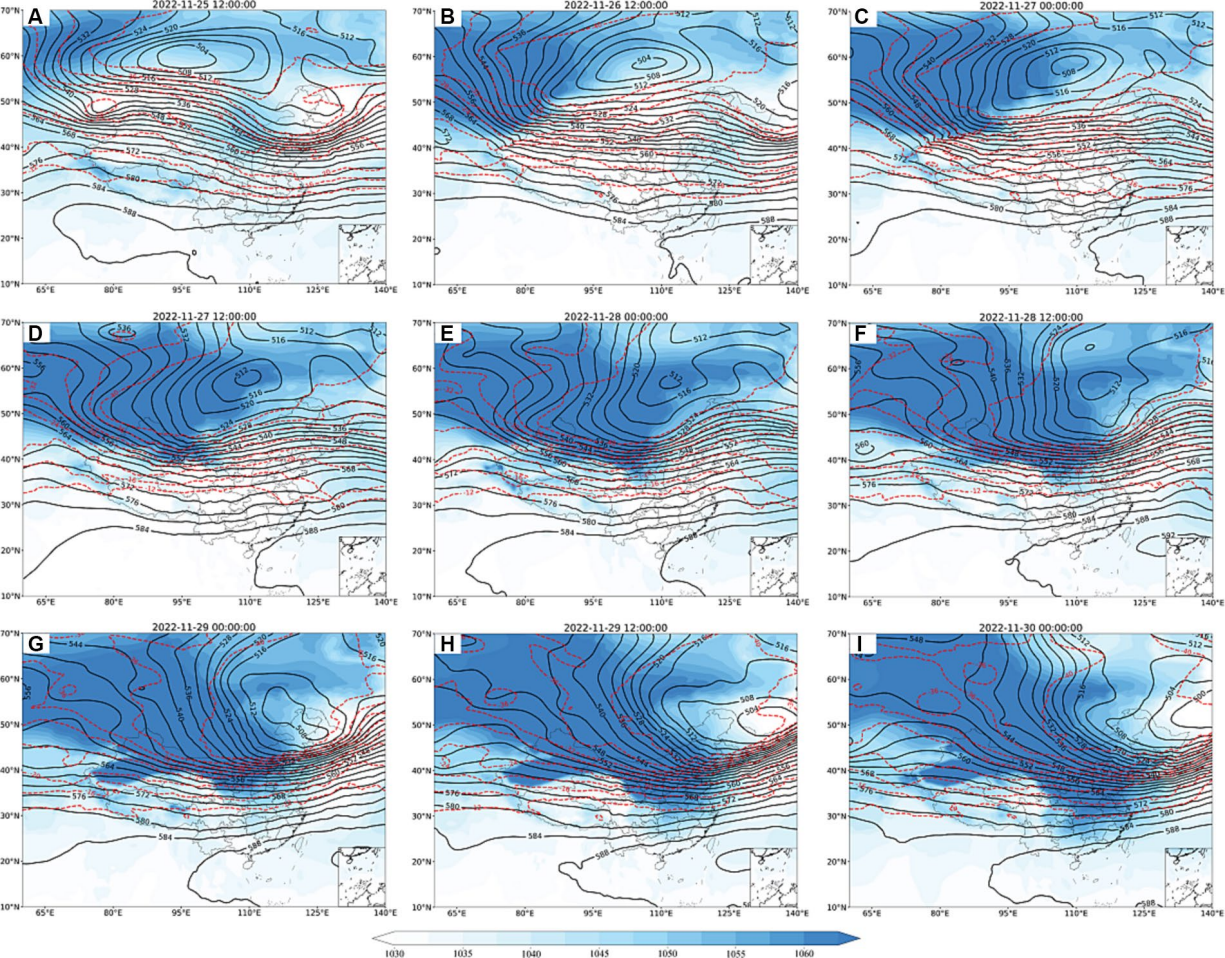
Geopotential height at 500 hPa (black solid line, unit: dagpm) and temperature field (red dashed line, unit: °C) and sea level pressure field (shaded, unit: dagpm; **A–I**) from 12:00 on 25 November to 00:00 on 30 November 2022.

As indicated in Figure 2, at 12:00 on 25 November, a circulation pattern manifested in the 500 hPa height field, revealing the presence of two ridges and a trough. This configuration established an inverted Ω-flow pattern over East Asia. Concurrently, a deep closed cold low-pressure system developed in the northwestern vicinity of Lake Baikal. Additionally, an extensive east–west oriented cross trough was observed in the northwestern region. The persistent transport of cold advection preceding the ridge positioned behind the trough fostered the accumulation of cold air within the cross trough. The intensified development of the blocking high located on the eastern side of the Ural Mountains, combined with the surface sea level pressure field can be seen behind a high altitude trough with negative vorticity advection behind the trough, which promoted the development of cold high pressure at the surface. By 00:00 on the 27th, the cross trough has moved to northern Xinjiang, China. Subsequently, the cold front advances towards the northern territories of Inner Mongolia and eastern Xinjiang. The cold front undergoes further intensification and propagates southeastward, thereby exerting its influence over a significant portion of northern regions within China. At 12:00 on the 28th, a notable transformation occurred as the previously established inverted Ω-flow pattern ceased to persist. This alteration was accompanied by the emergence of an outburst of cold air and a vertical reorientation of the horizontal trough. Therefore, a discernible meridional circulation pattern manifested in the wake of the trough. These atmospheric adjustments created favorable circumstances for its continued southward displacement. As the high-altitude fronts persistently advance towards the south, the cold front has extended its influence to encompass the central region of China by 00:00 on the 29th, then it reached further into southern regions of China, with particular emphasis on areas encompassing Hunan and Jiangxi provinces.

### 3.3 Assessment of mortality risk in the cold spell

**Figure 3:**
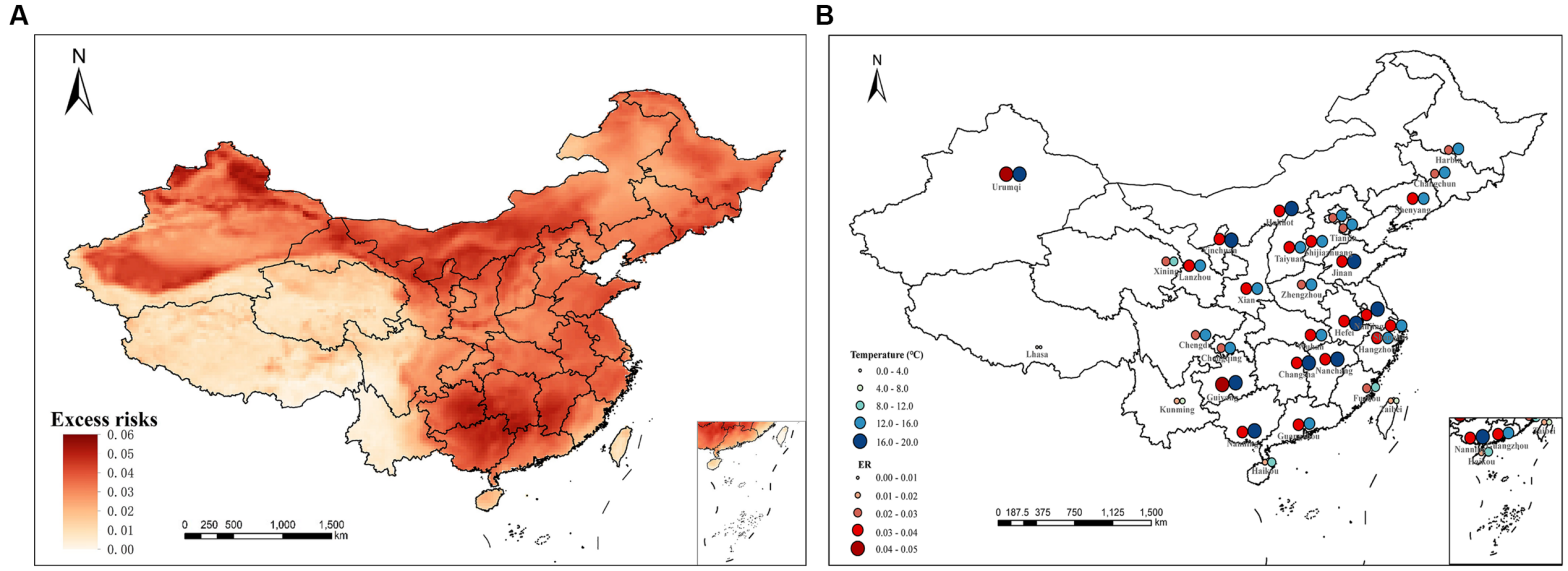
Distribution of excess risks **(A)** and the temperature drops and ERs in provincial capital cities in China **(B)**.

Figure 3A presents the spatial distribution of the excess risks (ERs) for the cold spell process. The spatial distribution of ER reveals a notable discrepancy in different regions. It pronounced high ERs in the northern and southern regions, and relatively lower ERs in the central areas. Specifically, regions exhibiting higher ERs include northwestern regions of China such as northern Xinjiang, central and western Inner Mongolia, northeastern Gansu, Ningxia, Shaanxi, northern Shanxi, as well as some southern regions such as eastern Guizhou, Hunan, southern Jiangxi, and northern Guangxi. Additionally, it is noteworthy that these identified areas are concurrently characterized by significant cooling. In Figure 3B, the ERs of provincial capital cities exhibit a distinct correlation with the low temperatures. This correspondence underscores the relationship between the severity of temperature decrease and the associated health risks. Among these cities, the highest ER is recorded in Urumqi (0.0435), followed closely by Guiyang (0.0424). Among the cities counted, only three recorded temperatures below 10°C, while the southern cities experienced significant temperature declines, accompanied by high ERs. More than 90% of southern cities had ERs greater than the average value of whole country. Combined with Figures 2, 3, it can also be observed that the temperature drops aligns with the movement of cold fronts. On November 26th, a ground cold front hit northern Xinjiang. The next day, it reached the southern part of Xinjiang and moved southeast, reaching central and western Inner Mongolia, Gansu, east-central, and northern Qinghai. On November 28th, the cold front had arrived in northern China, extending to northeast regions and Huanghuai. By November 29th, it had reached Sichuan and Chongqing on the west, and extended ulteriorly its reach the coast of South China on the 30th.

**Figure 4:**
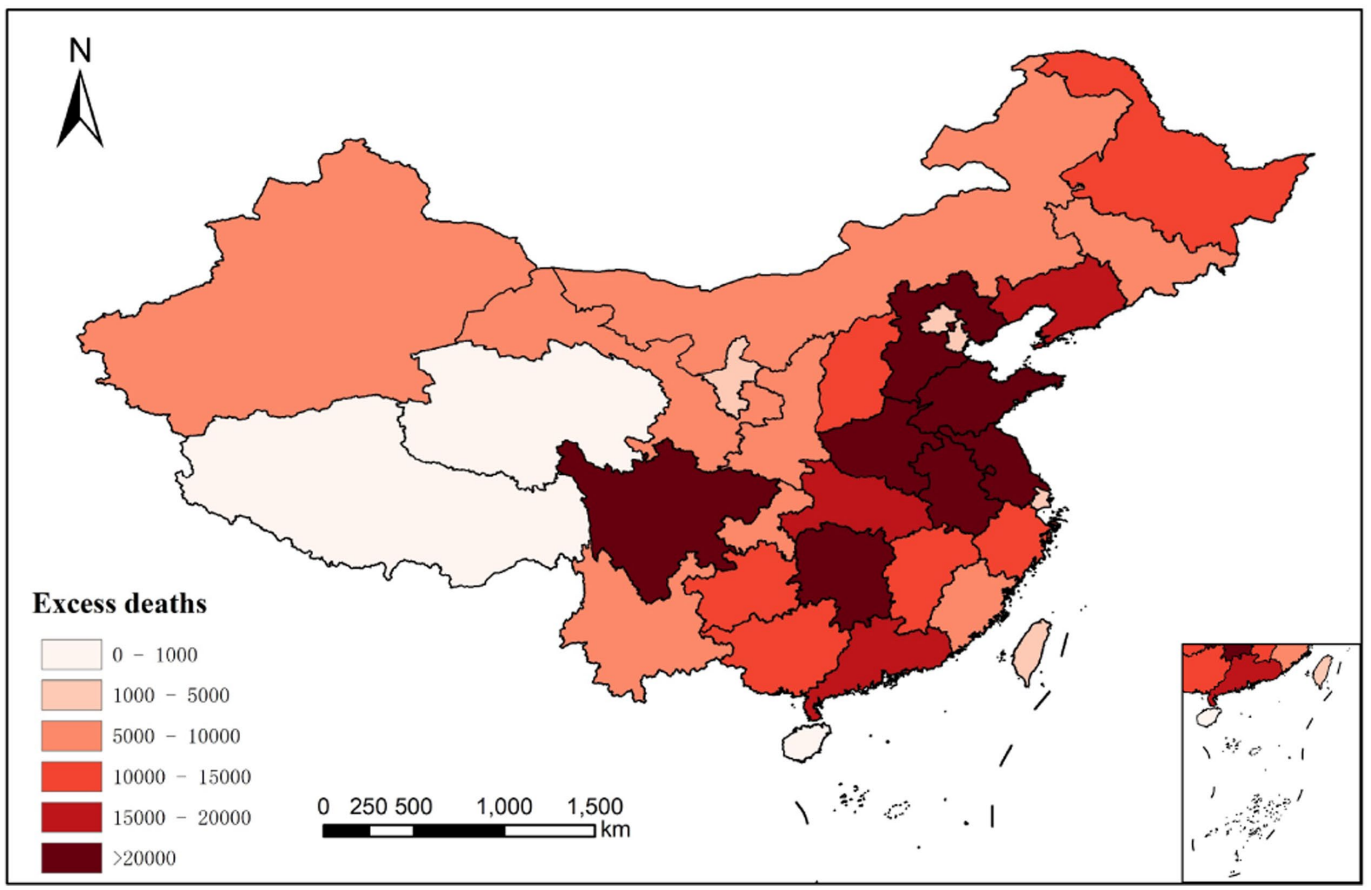
Excess deaths in China.

Figure 4 demonstrates the spatial distribution of cumulative excess deaths by province of China. The highest number of cumulative excess deaths was identified in Shandong with 29,492 cases, exemplifying the severity of the impact seen in this particular region. Conversely, Tibet exhibited the lowest number of cumulative excess deaths, with a recorded count of 196. The cumulative excess deaths exceeding 20,000 predominantly cluster within the central-eastern regions of China. Shandong Province emerges as the most heavily impacted province, followed by Hunan, Henan, Anhui, Sichuan, Hebei, and Jiangsu provinces. The provinces showcasing cumulative excess deaths ranging from 10,000 to 20,000 are situated predominantly in the southern and northeastern parts of the country, namely Hubei, Guangdong, Jiangxi, Guizhou, Heilongjiang, and Liaoning. In contrast, Tibet and Qinghai provinces in Tibetan Plateau have reported a comparatively lower cumulative number of excess deaths, below 1,000. Northern provinces of China, for the most part, registered cumulative excess deaths in the range of 5,000 to 10,000. Thus, regional disparities in excess mortality become evident when considering the distribution of cumulative excess deaths across various provinces in China. Despite having relatively smaller populations, the regions of Xinjiang and Inner Mongolia in China exhibit a higher risk of excess deaths and cumulative excess deaths. This can be attributed to the pronounced cooling effect prevalent in these areas.

Table 2 provided an overview of the average ERs for each individual province and whole country. The average ER for China was found to be 2.75%, indicating that for every 1°C drop in average daily temperature, the number of deaths is expected to increase by 2.75%. The excess risks of provinces unveiled both the highest and lowest values, recorded as 5.72 and 0.14%, respectively. Furthermore, a substantial 81.25% of provinces exhibit ERs surpassing the average value for whole country. Among the provinces, the top five provinces displaying the highest excess mortality risks are identified as Hunan (4.18%), Guangxi (4.17%), Guizhou (4.09%), Jiangxi (4.06%), and Ningxia (3.89%). These findings align with numerous prior studies indicating a greater influence of cold spells on southern regions of China compared to the northern areas.

**Table 2:** Average excess risks and excess deaths per kilometre in different provinces of China.

Provinces	Average ERs	Excess deaths per km^2^
Tibet	1.03%	0.0002
Xinjiang	3.01%	0.0045
Inner Mongolia	3.44%	0.0078
Gansu	3.17%	0.0169
Qinhai	1.29%	0.0013
Ningxia	3.89%	0.0291
Sichuan	1.79%	0.0459
Shaanxi	3.30%	0.0462
Shanxi	3.46%	0.0771
Chongqing	3.00%	0.0896
Yunnan	1.22%	0.0167
Guizhou	4.09%	0.0592
Guangxi	4.17%	0.0611
Henan	3.06%	0.1448
Hubei	3.33%	0.0935
Hunan	4.18%	0.1230
Guangdong	3.36%	0.0962
Hebei	3.32%	0.1072
Beijing	2.88%	0.2376
Tianjin	2.89%	0.1566
Shandong	3.54%	0.1878
Anhui	3.61%	0.1626
Jiangsu	3.57%	0.1976
Zhejiang	3.47%	0.1175
Jiangxi	4.06%	0.0756
Fujian	2.79%	0.0486
Shanghai	3.03%	0.5091
Heilongjiang	3.25%	0.0280
Jilin	3.23%	0.0466
Liaoning	3.26%	0.1159
Hainan	1.69%	0.0270
Taiwan	1.15%	0.0536
Average	2.75%	0.0381

**Figure 5:**
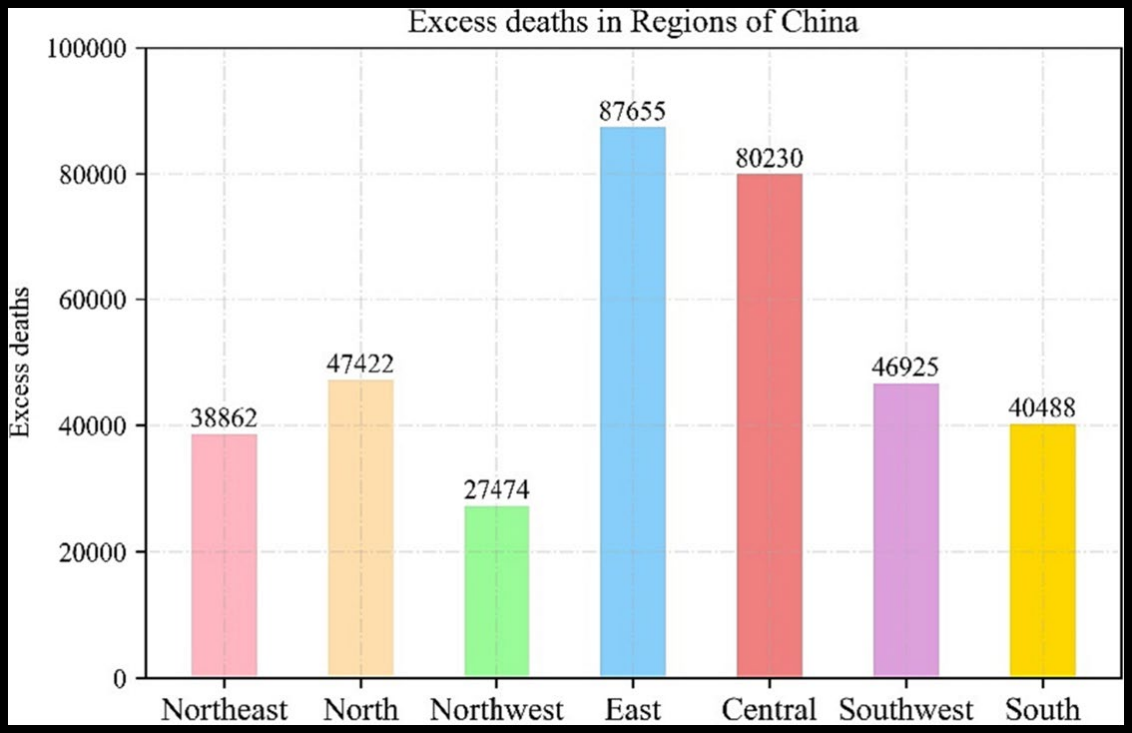
Excess deaths in seven geographical regions of China.

Figure 5 showed the cumulative excess number of deaths recorded across the seven distinct geographical regions within China. According to the statistics of cumulative excess deaths in each region, Eastern China emerges as the region with the highest fatality count, totaling 80,000 deaths. It is closely followed by central China, which also recorded 80,000 cumulative excess deaths. In contrast, Northwest China exhibited the lowest number of fatalities among the regions, while the North, South, and Southwest regions reported figures exceeding 40,000 deaths. Additionally, we conducted an analysis of cumulative excess deaths per square kilometer across the regions (see Table 2). Notably, Shanghai, situated in the eastern region, exhibited the highest density of cumulative excess deaths, with a value of 0.5091 deaths per square kilometer. In contrast, Tibet recorded the lowest density, with a mere 0.0002 deaths per square kilometer. The top five regions with the highest rankings in terms of cumulative excess deaths per square kilometer also include Beijing (0.2376), Jiangsu (0.1976), Shandong (0.1878), and Anhui (0.1626). The region with the most substantial cumulative excess deaths per square kilometer is Eastern China, a phenomenon closely associated with its densely populated areas and heightened vulnerability to the cold spells.

## 4 Discussion

From a circulatory perspective, the occurrence of the cold spell is primarily associated with the establishment and subsequent collapse of middle and high latitude blocking high pressure systems. The principle is by the eastward progression of an intensifying cold high-pressure system, resulting in the influx of strong cold air. The cyclonic nature of the cold high-pressure front experiences rapid intensification, leading to the development of a horizontal trough that eventually transforms into a vertical structure. This vertical alignment facilitates the intrusion of cold air into China, ultimately triggering the outbreak of a cold spell. Extensive experience and research have enabled the classification of short-medium term cold spell weather patterns into three main types: small trough development, eastward-moving low trough, and horizontal trough turning vertical (22). It should not be disregarded that the substantial human health consequences stemming from extensive nationwide cold spells. The impacts of population vulnerability related to these cold events are anticipated to become increasingly severe under future warming scenarios (23).

Previous studies reported the possible mechanisms of influence of cold air and dramatic temperature changes during cold spells on health, especially for respiratory and circulatory diseases (18, 24–26). It is noteworthy that low temperature exerts a more pronounced impact on mortality associated with respiratory diseases (17, 27). Cold temperatures can promote the survival of bacteria and viruses in droplets, and indoor overcrowding can contribute to an increased risk of transmission among individuals (28). Abrupt temperature drops can also compromise the local defenses of the human respiratory tract, giving rise to the development of various lung ailments (29–31). Under the circumstance of sustained hypothermia and the passage of a cold front, there is an increase in blood supply and circulatory load on the human heart and brain, resulting in elevating the incidence of hypertension, and consequently raising the risk of stroke (32, 33). The activity of cold air masses and changes in meteorological factors can induce or exacerbate respiratory and circulatory diseases with a certain degree of lag (34, 35). The impacts caused by these environmental factors may not be fully recovered within a short period of time (36). Especially in vulnerable populations such as children and the older adults, their thermoregulatory systems often struggle to adapt to persistent cold temperature and drastic temperature changes (37), which may lead to the development and exacerbation of related diseases (38).

Cold fronts and cold high pressure are both weather systems controlled by cold air masses, with a cold front situated along the front of the cold high pressure. As the systems move, meteorological elements can change drastically, particularly leading to short periods of substantial cooling and rapid increases in barometric pressure (22). In the current study, we observed that such alterations in temperature and pressure caused by the movement of cold fronts often resulted in high ERs. Several studies have investigated the intricate relationship between mortality and changes in temperature and pressure, generally revealing an augmented mortality risk linked to elevated temperatures and reduced pressure (39–41). In a research conducted in the Czech Republic, changes in meteorological elements were found to be significantly correlated with mortality rates. The relationship between excess mortality and changes in temperature and pressure was more pronounced in instances of sudden fluctuations than in the passage of atmospheric fronts, and the effects were observed predominantly in populations aged 70 years and older (42). Similarly, Morabito et al. (43) identified a connection between abrupt weather changes and heightened blood pressure levels in Italy. Moreover, several other studies conducted in UK and the United States of America have also highlighted the association between sudden changes in weathers and mortality (44, 45).

Our findings indicated that the average ERs of death were greater in southern provinces of China compared to northern provinces. This discrepancy may be attributed to the differential adoption of protective measures by the public in these respective regions during cold spell events. The inhabitants of northern China exhibit a higher propensity for employing effective protective measures, including the provision of indoor heating in households and the implementation of community adaptation strategies. Individuals residing in warmer regions often face heightened vulnerability to the impacts of cold weather due to their limited physiological and behavioral adaptations (46–48). For instance, in subtropical regions, the scarcity of buildings equipped with heating systems capable of providing adequate warmth during extremely cold weather conditions contributes to an amplified risk, particularly among vulnerable groups such as the older adults (49). Conversely, northern regions exhibit greater adaptive experience with regard to lifestyle, dietary habits, clothing choices, and internal mechanisms that help regulate the body’s response to cold spell exposure (50). Individuals from disadvantaged socio-economic backgrounds and with lower levels of education are often more vulnerable to the effects of cold due to their limited access to health services and poorer living conditions (18, 51). When coupled with their geographical location in the warmer southern region, these factors render them even more susceptible to the adverse effects of lower temperatures (19, 52, 53). However, Shandong has the highest cumulative excess deaths. This may be due to the calculation of cumulative excess deaths accounts for various factors, including baseline mortality rates, population, and temperature drops during cold spells. Shandong Province is a populous region in China, ranking second in terms of population according to the seventh national census, with a relatively high baseline mortality rate. Additionally, the majority of the province experienced a significant decrease in temperature during the cold spell. Other provinces such as Xinjiang and Inner Mongolia had significant cooling but sparse populations, while Guangdong and Henan had large population bases but did not experience as much overall temperature reduction as Shandong. Consequently, the comprehensive result made Shandong have the highest cumulative excess deaths.

It is significant to note the potential higher vulnerability of early winter cold events examined in this study. Previous studies have shown that the risk associated with short-term, early cold and extreme cold events is higher compared to late cold events, with longer duration of early cold events exhibiting greater vulnerability (54, 55). Nevertheless, relative risks decrease towards the latter part of winter, which may be related to people gradually adapting to the cold environment by taking appropriate warming measures. The health risks associated with the first cold spell are greater compared to previous studies. Sun et al. (19) found that 57,783 non-accidental deaths were related to the cold spell in China in 2018, while our analysis revealed a significantly higher cumulative excess mortality of 369,056. In terms of attributing the excess mortality risk associated with cold spells, the national average ER (2.75%) estimated in our study was higher than 2.10% for multiple cities in China (56) and 1.44% in South Korea and Japan (57). This implies that the first cold spell process may have a stronger harvest effect (58), which may have a greater impact on public health in the short term, particularly among individuals with underlying medical conditions. Furthermore, the cold spell in this study exhibited a protracted duration, significant intensity, and diverse spectrum of ramifications. While the intensity and duration are crucial factors influencing the cold mortality relationship, with longer and stronger cold spells being associated with a significantly increased mortality rate (59, 60). Within the context of global climate change, it has been observed that wherein cold spells are becoming less frequent but increasingly extreme in nature (61). A preceding study suggested a notable escalation in the risk and health burden associated with cold spells over the course of several decades (57). It is crucial that we should keep moving forward assessing health risks in connection with cold waves and predicting the future health impacts of climate change, and comprehend the necessity of the intricate interplay between frigid weather events and human health. Simultaneously, we also should underscore the need for proactive measures to mitigate the impacts of extreme weather events such as cold waves and reduce the burden of disease on vulnerable populations.

Several limitations should be acknowledged in this study. Firstly, our estimates are subject to the assumption that a uniform baseline mortality rate is applied to each locality within the province. This simplifying assumption may overlook potential variations in mortality rates across different sub-regions or demographic groups within each province. Secondly, the data utilized for establishing the baseline mortality rates were only updated until 2021, potentially limiting the generalizability of our findings to more recent periods. Considerable variations in mortality rates arise between urban and rural areas owing to disparities in socio-economic levels, environmental factors, and access to healthcare services (62). We have employed a single β value for China, which may lead to potential underestimation or overestimation of the health burden in certain areas. Significant demographic and epidemiological changes were driven by rapid urbanisation, new coronavirus epidemics, and ageing (63), but we were unable to acquire the exact number of deaths caused by cold wave exposure to facilitate the calculation and validation processes due to data constraints. Future studies could benefit from incorporating updated health data, which would enhance the precision and reliability of studies.

## 5 Conclusion

The findings indicated that temperature drops from the cold spell outbreak had placed a significant health burden on China. Southern China faced a greater health risk from the cold spell compared to the north. The economically developed and densely populated provinces and regions have a higher cumulative excess deaths. Excess deaths were the highest in Shandong province. Our research offered an evidence of the mortality risks posed by cold spells in China, and may play a crucial role in developing region-specific prediction systems, particularly vulnerable groups, from the hazards of these extreme weather events.

## Data availability statement

The original contributions presented in the study are included in the article/Supplementary material, further inquiries can be directed to the corresponding author.

## Author contributions

WW: Methodology, Writing – original draft. YM: Conceptualization, Methodology, Writing – review & editing. PQ: Data curation, Formal analysis, Writing – original draft. ZL: Formal Analysis, Writing – original draft. YZ: Validation, Writing – original draft. HJ: Software, Writing – original draft.

## Footnotes

^1^ https://data.cma.cn

^2^ https://www.resdc.cn

^3^ https://data.stats.gov.cn/

^4^ http://www.cma.gov.cn
