# Supplementary material for: Assessment of mortality risks due to a strong cold spell in 2022 in China
Source: Front Public Health. 2023 Dec 7;11:1322019. doi: 10.3389/fpubh.2023.1322019 (PMC10733490; doi:10.3389/fpubh.2023.1322019)
Supplement: Supplementary file 1 [file Table_1.DOCX]

**Supplementary Material**

**Table S1** Statistics of baseline mortality and natural growth rate of population from 2020 to 2022 by province in China

| Provinces | Baseline Mortality rate (‰) | Natural growth rate of population in 2020 (‰) | Natural growth rate of population in 2021 (‰) | Natural growth rate of population in 2022 (‰) |
| --- | --- | --- | --- | --- |
| Tibet | 5.47 | 8.59 | 8.70 | 8.76 |
| Xinjiang | 5.6 | 1.48 | 0.56 | 0.77 |
| Inner Mongolia | 7.54 | -0.10 | -1.28 | -2.25 |
| Gansu | 8.26 | 2.64 | 1.42 | -0.04 |
| Qinhai | 6.91 | 4.78 | 4.31 | 3.37 |
| Ningxia | 6.09 | 5.71 | 5.53 | 4.41 |
| Sichuan | 8.74 | -0.88 | -1.89 | -2.65 |
| Shaanxi | 7.38 | 1.84 | 0.51 | -0.28 |
| Shanxi | 7.32 | 1.24 | -0.26 | -0.98 |
| Chongqing | 8.04 | -0.23 | -1.55 | -2.11 |
| Yunnan | 8.12 | 3.04 | 1.23 | -0.07 |
| Guizhou | 7.19 | 6.53 | 4.98 | 3.71 |
| Guangxi | 6.8 | 4.90 | 2.88 | 1.43 |
| Henan | 7.36 | 2.09 | 0.64 | -0.08 |
| Hubei | 7.86 | 0.61 | -0.88 | -2.01 |
| Hunan | 8.28 | 0.61 | -1.15 | -2.31 |
| Guangdong | 4.83 | 5.58 | 4.52 | 3.33 |
| Hebei | 7.58 | 0.94 | -0.43 | -1.71 |
| Beijing | 5.39 | 1.80 | 0.96 | -0.05 |
| Tianjin | 6.23 | 0.07 | -0.93 | 1.43 |
| Shandong | 7.36 | 1.31 | 0.02 | -0.93 |
| Anhui | 8.00 | 1.49 | 0.05 | -0.93 |
| Jiangsu | 6.77 | 0.16 | -1.12 | -1.81 |
| Zhejiang | 5.90 | 0.57 | 1.00 | 0.04 |
| Jiangxi | 6.71 | 2.87 | 1.63 | 0.25 |
| Fujian | 6.28 | 2.97 | 1.98 | 0.55 |
| Shanghai | 5.59 | -0.56 | -0.92 | -1.61 |
| Heilongjiang | 8.70 | -4.48 | -5.11 | -5.75 |
| Jilin | 8.08 | -2.97 | -3.38 | -4.07 |
| Liaoning | 8.89 | -3.43 | -4.18 | -4.96 |
| Hainan | 6.01 | 4.51 | 3.73 | 2.44 |
| Taiwan | 7.86 | -0.34 | -1.28 | -2.93 |

**Table S2** ERs in major cities of China

| Cities | Excess Risks |
| --- | --- |
| Changji | 4.17% |
| Turpan | 4.82% |
| Tianshui | 4.76% |
| Dingxi | 3.85% |
| Fuyang | 2.92% |
| Nyingchi | 3.42% |
| Chifeng | 4.77% |
| Haidong | 3.16% |
| Wuzhong | 3.71% |
| Weinan | 3.26% |
| Shenzhen | 2.59% |
| Yulin | 3.44% |
| Bijie | 3.09% |
| Qionghai | 4.64% |
| Nanyang | 4.07% |
| Huanggang | 3.34% |
| Hengyang | 4.34% |
| Suzhou | 3.20% |
| Qingdao | 3.92% |
| Yuncheng | 2.85% |
| Nanchong | 2.83% |
| Qujing | 2.01% |
| Xiamen | 1.59% |
| Qiqihar | 2.37% |
| Jilin | 3.45% |
| Dalian | 3.08% |
| Dongguan | 2.98% |
| Ningbo | 3.59% |
| Foshan | 3.21% |
| Wuxi | 3.32% |


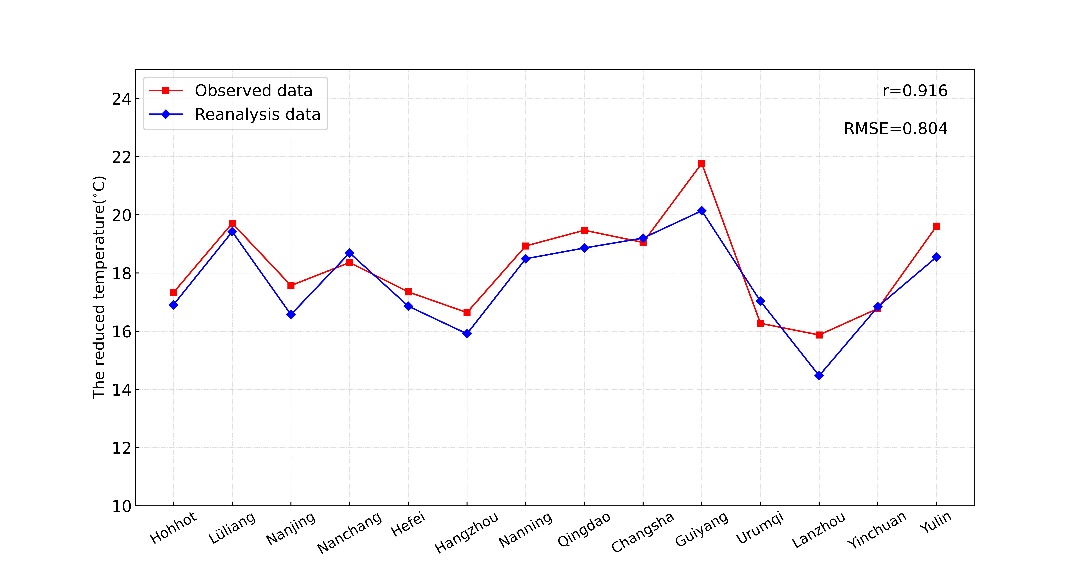


**Fig. S1** Comparison of data from observation stations in 14 major cities with ERA5 reanalysis data (correlation coefficient: r, root mean square error: RMSE)

The formulas are shown below:

$$r=\frac{\Sigma_{i=1}^{n}(T_{o,i}-\bar{T}_{o,i})\cdot(T_{r,i}-\bar{T}_{r,i})}{\sqrt{\Sigma_{i=1}^{n}(T_{o,i}-\bar{T}_{o,i})^{2}}\cdot\sqrt{\Sigma_{i=1}^{n}(T_{r,i}-\bar{T}_{r,i})^{2}}}$$

$RMSE=\sqrt{\frac{1}{n}\sum_{i=1}^{n} \left(T_{o,i}-T_{r,i}\right)^{2}}$

where $T_{o,i}$ is the temperature reduction of cold spell at the observation station and $T_{r,i}$ is the temperature reduction of process for the ERA5 reanalysis data.
